# Inhibition of Lipopolysaccharide- and Lipoprotein-Induced Inflammation by Antitoxin Peptide Pep19-2.5

**DOI:** 10.3389/fimmu.2018.01704

**Published:** 2018-07-26

**Authors:** Lena Heinbockel, Günther Weindl, Guillermo Martinez-de-Tejada, Wilmar Correa, Susana Sanchez-Gomez, Sergio Bárcena-Varela, Torsten Goldmann, Patrick Garidel, Thomas Gutsmann, Klaus Brandenburg

**Affiliations:** ^1^Clinical and Experimental Pathology, Research Center Borstel, Borstel, Germany; ^2^Institute of Pharmacy (Pharmacology and Toxicology), Freie Universität Berlin, Berlin, Germany; ^3^Department of Microbiology and Parasitology, Universidad de Navarra, Pamplona, Spain; ^4^Biophysics, Research Center Borstel, Borstel, Germany; ^5^Martin-Luther Universität Halle-Wittenberg, Halle, Germany

**Keywords:** antimicrobial peptides, Pep19-2.5, sepsis, intracellular LPS signaling, endotoxin

## Abstract

The most potent cell wall-derived inflammatory toxins (“pathogenicity factors”) of Gram-negative and -positive bacteria are lipopolysaccharides (LPS) (endotoxins) and lipoproteins (LP), respectively. Despite the fact that the former signals *via* toll-like receptor 4 (TLR4) and the latter *via* TLR2, the physico-chemistry of these compounds exhibits considerable similarity, an amphiphilic molecule with a polar and charged backbone and a lipid moiety. While the exterior portion of the LPS (i.e., the O-chain) represents the serologically relevant structure, the inner part, the lipid A, is responsible for one of the strongest inflammatory activities known. In the last years, we have demonstrated that antimicrobial peptides from the Pep19-2.5 family, which were designed to bind to LPS and LP, act as anti-inflammatory agents against sepsis and endotoxic shock caused by severe bacterial infections. We also showed that this anti-inflammatory activity requires specific interactions of the peptides with LPS and LP leading to exothermic reactions with saturation characteristics in calorimetry assays. Parallel to this, peptide-mediated neutralization of LPS and LP involves changes in various physical parameters, including both the gel to liquid crystalline phase transition of the acyl chains and the three-dimensional aggregate structures of the toxins. Furthermore, the effectivity of neutralization of pathogenicity factors by peptides was demonstrated in several *in vivo* models together with the finding that a peptide-based therapy sensitizes bacteria (also antimicrobial resistant) to antibiotics. Finally, a significant step in the understanding of the broad anti-inflammatory function of Pep19-2.5 was the demonstration that this compound is able to block the intracellular endotoxin signaling cascade.

## Introduction

Peptide-based therapies for diverse applications are under investigation since many years. Particularly, antimicrobial peptides (AMPs) are a subject of considerable research with some particular compounds reaching clinical use. Further development, improvement, and expansion for new microbial targets make the understanding of the underlying molecular mechanisms mandatory. AMPs have a wide range of therapeutic activities depending on their mode of action on the target structures. The main strategies include (i) to optimize their direct antimicrobial activity (i.e., lowering their minimal inhibitory and minimal bactericidal concentrations—MIC and MBC values), (ii) to provide an immunomodulatory activity (i.e., enhancing the human defense system), and (iii) to bind to the responsible immune-stimulating compounds of bacteria, lipopolysaccharide, and lipoproteins, and thus to inhibit inflammation. Approach (i) led to the investigation and development of some antibacterial drugs, with the disadvantage that with increasing broad-spectrum effect also the hemolytic activity and other side effects increased. The development of approach (ii) led to some relevant insights into immune processes, but to no successful clinical application, whereas strategy (iii) is increasingly being considered as a highly interesting therapeutical option and is in the focus of the present review.

Finally, a pivotal goal in the development of AMPs is to broaden their spectrum of antibacterial and anti-inflammatory activity while ensuring low toxicity against host cells. This balancing act has to be carefully adjusted and this will also be discussed in this work.

## Essential Action of AMPs

A critical issue in the treatment of infectious disease is the high or even chronic inflammatory status of the patients. The release of toxins from the bacterial cell wall due to upregulation of immune system effectors or to an antibiotic therapy often contributes to worsen the final outcome (1). Therefore, it is of crucial interest during antibiotic treatment not only to kill the bacteria but also to prevent excessive inflammation mediated by cell debris released by lysed bacteria. Some well-known antibiotics of the polymyxin AMP family (cyclic lipopeptides active against Gram-negative bacteria) combine a potent bactericidal activity with the capacity to efficiently neutralize endotoxins. Regrettably, due to their propensity to cause undesirable side effects such as neuro- and nephrotoxicity, they are used as drugs of last resource (2).

**Figure 1:**
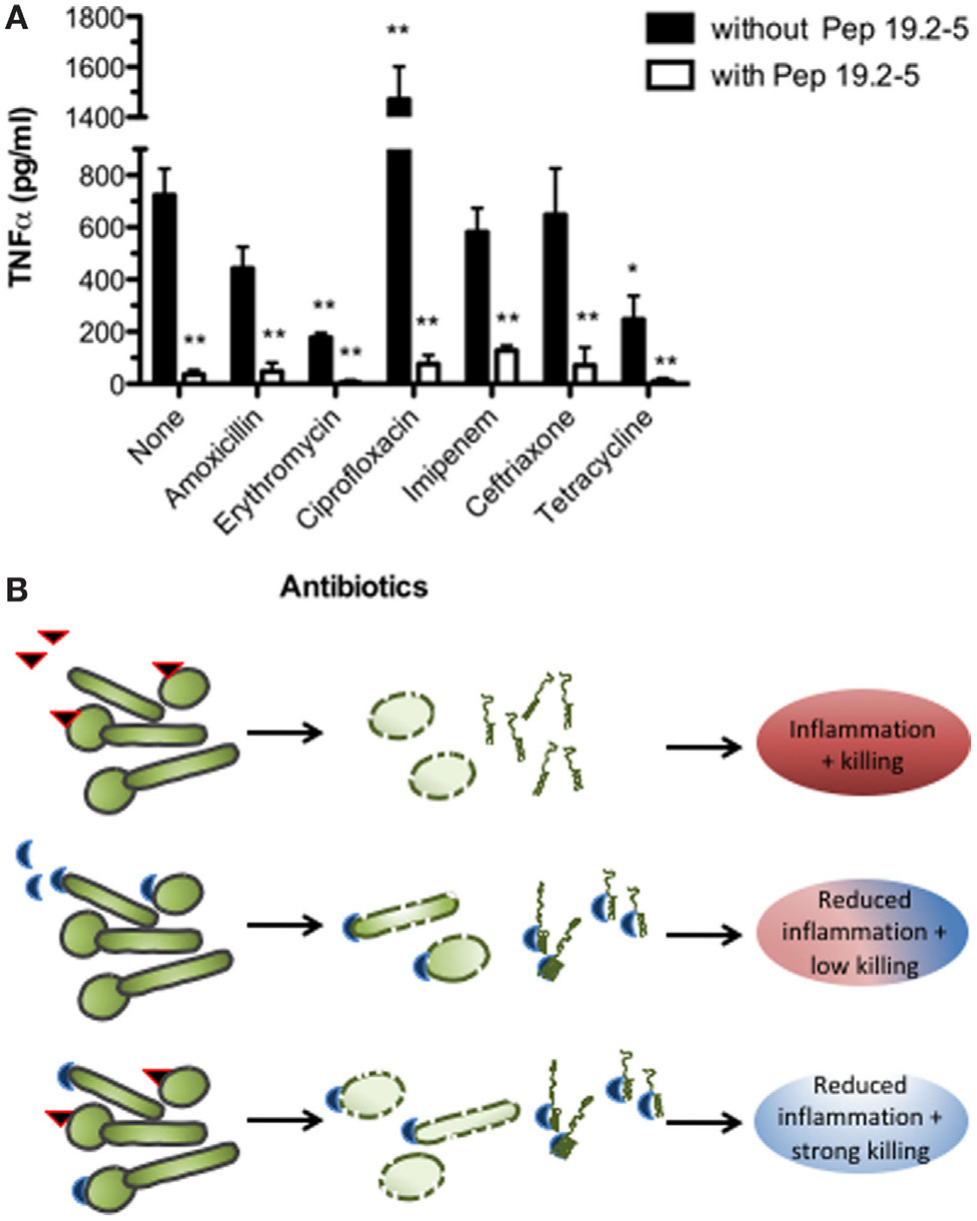
Antimicrobial and anti-inflammatory therapies. **(A)** Therapeutic efficacy of Pep19-2.5 combined with antibiotics to control bacteremia and endotoximia caused by intraperitoneally injected *Salmonella enterica* sv. Minnesota (10^7^ cfu/mouse). Bacteremia was treated either with various antibiotics or antibiotics plus Pep19-2.5 and the TNFα serum levels were measured 90 min after bacterial challenge and treatment (4). **(B)** Antibiotics (red triangle) kill the bacteria, while Pep19-2.5 (blue semicircle) destabilizes the bacterial membranes and neutralizes the toxins. As presented in the last row, the peptide may bind to the toxins as constituents of the bacteria or in isolated form, thus inhibiting the strong inflammation reaction.

One of the most promising approaches for designing new AMPs is the use of LPS-binding polypeptide domains within defense proteins such as lactoferrin and *Limulus* anti-LPS-factor (LALF). This has successfully been done in previous studies [for an overview, see Ref. (3)]. Using these domains as templates and performing a rational design focused on optimizing their lipid A-binding and neutralizing activity, we developed the Aspidasept^®^ family of compounds (also called SALP, synthetic anti-LPS peptides). Within this family, Pep19-2.5 and Pep19-4LF are undergoing preclinical testing. Although these polypeptides exhibit a more modest antimicrobial activity against Gram-negative bacteria compared to polymyxin B, they are endowed with a remarkable capacity to kill Gram-positive bacteria. In addition, these AMPs have an increased ability to neutralize toxins from both type of organisms, namely lipopolysaccharides (LPS) and lipoproteins (LP) (4, 5). Interestingly, we demonstrated that Pep19-2.5 efficiently counteracts the pro-inflammatory activity of some antibiotics such as ciprofloxacin and ceftriaxone [Figure 1 and Ref. (6), respectively] and cooperates *in vivo* with several structurally unrelated antibiotics to neutralize serum levels of TNF-alpha induced by a bacterial infection (Figure 1). Therefore, a combined medication based on antibiotics and toxin-neutralizers offers great promise for the treatment of patients with inflammatory diseases caused by bacterial infections, such as sepsis.

In addition to peptides from Aspidasept, polymyxin, or lactoferrin families, other interesting compounds are under current investigation. Thus, for example, the well-known human cathelidicin LL-37 is a peptide with multiple biological activities including the potential to act as an anti-endotoxin, immunomodulatory, and wound-healing compound (7). This peptide has the capacity to kill Gram-negative and Gram-positive bacteria, and it is able to neutralize endotoxin by sequestering soluble LPS (7–9). It was found that its activity against bacteria is in close relationship to its immunomodulatory function (4). The potency of this peptide to kill bacteria was found to be lower than that of polymyxin B or Pep19-4.LF, but it has similar LPS neutralizing ability compared to polymyxin B and Pep19-2.5 derivatives. Another interesting AMP is the cecropin d-like peptide (Gm1), a non-cationic AMP from *Galleria mellonella*. Gm1 was shown to have a broad spectrum of antimicrobial activity and may represent a good template for the peptide-based drug development of antisepsis compounds (10–12). The ability of this peptide to bind and neutralize LPS demonstrates that a polycationic character is not necessarily a prerequisite for an effective binding to LPS (11). This conclusion opens the prospect of developing a whole new peptide-based strategy to control sepsis.

## LPS/LP-Induced Inflammation

The basic mechanisms of LPS/LP-induced inflammation run *via* stimulation of cell-surface receptors on immune cells, toll-like receptor 4 (TLR4) for LPS and TLR-2 for LP, which subsequently leads to an intracellular reaction by recruiting transcription factors such as NF-κB in the nucleus followed by the secretion of chemokines and cytokines. In septic patients, this reaction gets out of control with the subsequent life-threatening “cytokine storm.”

Research in the field of sepsis prevention, but also of other serious infection-triggered inflammations, has been plagued with many failed clinical trials (13, 14). A major reason for this could be the overly specific—and thereby, narrow spectrum of biological activities displayed by the drugs under development. Prominent examples include the monoclonal anti-LPS antibodies E5 (15) and H1-A1 (16), which failed to improve survival of septic patients in clinical Phase III trials. These compounds were selected by their ability to bind to a collection of different endotoxins. This apparently promising approach ignores the fact that there is an innumerable diversity of Gram-negative as well as Gram-positive pro-inflammatory PAMPs (i.e., pathogen-associated molecular patterns) inducing, often in parallel, sepsis. Thus, for therapeutic efficiency, AMPs need to display a broad spectrum of anti-inflammatory activity for multifaceted infections, as well as sufficient bactericidal activity. A peptide possessing this dual behavior against bacterial infections in several sepsis models but also in other disease models is Pep19-2.5.

Notably, Pep19-2.5 and Pep19-4.LF inhibit inflammatory responses triggered by LP and LPS and mediated by the pattern recognition receptors (PRRs), toll-like receptor 2 (TLR2) and TLR4, respectively. This has also been confirmed in skin cells including keratinocytes, dermal fibroblasts, and dendritic cells (17). Interestingly, an additional mode of action has been identified in keratinocytes. Both peptides accelerated already at low concentrations artificial wound closure and increased cell migration of keratinocytes *via* purinergic receptor activation. These findings are particularly relevant to bacterial skin infections, which are often associated with impaired wound healing.

**Figure 2:**
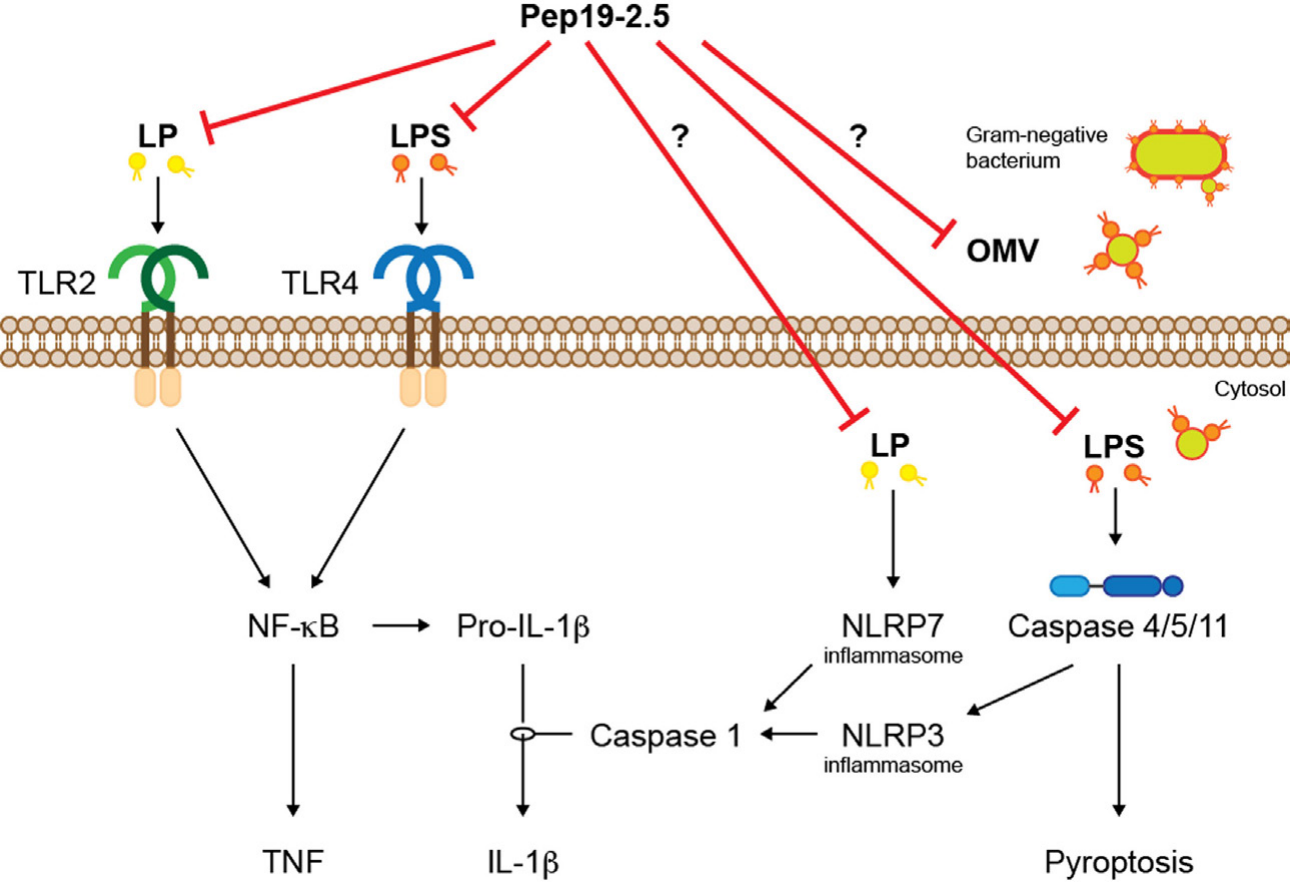
The synthetic peptide Pep19-2.5 inhibits signaling of LP and LPS mediated by transmembrane and cytosolic PRRs. The activated signaling cascades lead to inflammation and a form of cell death termed pyroptosis. LP, lipopeptides; LPS, lipopolysaccharides; OMV, outer membrane vesicle.

Besides transmembrane, PRRs, LPS, and LP are recognized intracellularly by cytosolic PRRs, which sense intracellular infections (Figure 2). The intracellular LPS sensor caspase-11 and its human orthologs caspase-4/5 cause activation of inflammasomes leading to production of IL-1β and cell death termed pyroptosis (18). The intracellular effects of LPS are thought to be crucial in the pro-inflammatory response during sepsis (19) and may at least partially explain the failure of TLR4 inhibitors in clinical trials. Given the neutralizing mode of action of SALPs, it is likely that the peptides inhibit not only extracellular TLR2/4 signaling but also intracellular signaling cascades mediated by inflammasomes. Indeed, Pep19.2-5 dampened intracellular LPS-induced caspase-1 activation, IL-1β production, as well as high mobility group box (HMGB)1 secretion and lactate dehydrogenase release in human cells (20). Although the underlying mechanisms are not fully understood, Pep19.2-5 may bind LPS extracellularly preventing its intracellular accumulation or translocate across the cell membrane and neutralize LPS intracellularly. A similar mode of action can be assumed for acetylated LP such as fibroblast-stimulating lipid 1 (FSL-1), which activates the NLRP7 inflammasome in the cytosol (21).

Since most Gram-negative bacteria that activate caspase-11/4/5 do not reach the cytosol, mechanisms are required that mediate internalization of LPS. Recent studies suggest that LPS is delivered into the cytosol *via* outer membrane vesicles (OMVs) that are secreted by Gram-negative bacteria (22). OMVs have been implicated in the pathogenesis of infectious diseases such as sepsis, thus, it would be interesting to know whether LPS-neutralizing peptides are able to interfere with OMVs to prevent intracellular LPS-mediated IL-1β production and pyroptosis.

## Biophysical Mechanisms of the Toxin-Neutralization Process

The interaction of LPS with Pep19-2.5 was investigated with a variety of physical techniques: (I) Fourier-transform infrared spectroscopy (FTIR) and differential scanning calorimetry to analyze the gel to liquid crystalline phase transition of the acyl chains of the toxins; (II) small-angle X-ray scattering (SAXS) with synchrotron radiation and freeze-fracture electron microscopy to determine their aggregate structure; (III) Zeta sizer analysis for surface charge and electrophoretic mobility determination; (IV) isothermal titration calorimetry for measuring the binding constants and saturation of the toxin:peptide complexes; and (V) Förster resonance energy transfer spectroscopy for intercalation experiments of the peptide into the toxin aggregates or target cell membranes (4, 23, 24).

The data on the gel to liquid crystalline phase transition showed a fluidization of the hydrocarbon chains of LPS from *Salmonella enterica* rough mutant chemotype R60 due to binding of Pep19-2.5. This phenomenon was detectable to a lesser extent in the FTIR experiment, but led to a complete disappearance of the phase transition in the calorimetric scan. In the former method, the fluidization could be deduced from the increase of the wave numbers of the symmetric stretching vibration of the methylene groups at 2,850 cm^−1^ in the gel phase below the phase transition temperature at around 36°C. Parallel to this increase, the change of the heat capacity between the two phases decreased considerably. Similar behavior was reported for the interaction of LPS with polymyxin B (25). Interestingly, the interaction of LPS with a peptidic portion of the human hemoglobin gamma-chain, called Hbγ35, led to a decrease in fluidization (i.e., a rigidification) of the LPS assembly (26). In contrast to the Pep19-2.5 series, the hemoglobin-derived peptide does not antagonize endotoxin activities upon binding to LPS but even enhances the LPS-induced cytokine secretion.

Investigations into the change of the aggregate structures of LPS due to peptide binding revealed a drastic reorientation of the LPS aggregates from a bilayered conformation, possibly in cubic symmetry, into a multilamellar arrangement (24). This could be proven in the SAXS experiments by the occurrence of reflections around 9.00 and 4.50 nm, which—as shown earlier—corresponds to the main reflections of LPS R60 in a multilayered assembly (27). These findings in the SAXS experiment were independently confirmed by freeze-fracture electron microscopy data, indicating stacks of membraneous arrangements of LPS with periodicities of the same size (9.0 nm) as found above. Similar results were reported also for the aggregate structures of wild-type LPS (from *E. coli* O55:B5 and *Salmonella abortus equi*) (28). Here, the binding of Pep19-2.5 to the latter LPS leads to a change from a mixed lamellar/non-lamellar aggregate structure into a multilamellar one with a periodicity of 9.20 nm. This value fits with that produced by the bioactive LPS Ra fraction (the rough LPS form) within the heterogeneous wild-type LPS. The observation that the endotoxically active unit within wild-type LPS corresponds to a Ra-LPS, was originally reported by Jiao et al. (29). Also here, the findings regarding multilamellarization events are in agreement with those reported for polymyxin B (30).

Data on the measurements of the electrophoretic mobility of LPS aggregates (Zeta potential) in the presence of peptides with either a potent (Pep19-2.5) or a weak (Pep19-8) LPS-neutralizing activity showed a compensation of the LPS head group charges by both peptides, with an even stronger action of the latter peptide (24). This is a clear indication that the neutralization of the negative charges within the LPS backbone is necessary but not sufficient for an effective anti-inflammatory action. These data could be convincingly confirmed by ITC measurements (4, 23), which showed binding of Pep19-2.5 to LPS as an exothermic process, which was saturated at a much lower molar ratio for Pep19-2.5 [(Pep19-2.5):(LPS) = 0.3], compared to Pep19-8 [(Pep19-8):(LPS) = 1.2]. This binding process can be explained as a two-step event consisting of a Coulomb interaction between the basic AA (R and K) with the negative charges of LPS (phosphates, carboxylates), followed by the hydrophobic interaction of the C-terminal region of the peptides (FWFWG) with the lipid A moiety of LPS. This interpretation is backed by the observation that a peptide variant (Pep19-2.5gek) lacking the C-terminal region was nearly unable to neutralize LPS, most likely because the second step of interaction could not take place. Pep19-2.5 was also reported to efficiently bind and neutralize a model toxin from a non-Gram-negative (mycoplasmic) bacteria, called FSL-1. Binding of the peptide to FSL-1 was associated with a strong exothermic reaction with saturation characteristic (31) similar as found for LPS.

Consistent with this explanation, the interaction of the inflammation-enhancing peptide Hbγ35 with LPS—although an exothermic process—exhibited no saturation characteristics (26).

To study the interaction of peptides with phospholipid bilayers, FRET spectroscopy was applied (24). It was found that selected peptides (i.e., Pep19-2.5, its scrambled version Pep19-2.5KO, and the compound with low LPS-neutralizing activity, Pep19-8), all intercalated readily into phosphatidylcholine and phosphatidylserine liposomes as well as into LPS aggregates. There was no correlation between the different LPS antagonistic activities of the peptides and their ability to interact with lipid bilayers. Similarly, when peptides were added to aggregates formed by amphiphilic molecules other than LPS, such as FSL-1 and the lipoprotein SitC from *S. aureus*, all SALPs incorporated readily (31).

Finally, binding of LPS to either the inflammation-inhibiting peptide Pep19-2.5 or to the inflammation-enhancing peptide Hbγ35 led to antagonistic results. Thus, whereas the former peptide caused an increase in aggregate size, connected with the adoption of a multilamellar structure, the latter decreased that parameter, probably coupled with the production of smaller bilayered LPS-aggregates with cubic symmetry (26).

## Conclusion

Peptides combine features that make them attractive candidates for the treatment of infectious and inflammatory diseases. On the one hand, they can be produced in high amounts using simple and affordable procedures. In addition, since AMPs consist of natural amino acids, these compounds are rapidly metabolized in the body without generating toxic by-products. Pep19-2.5 neutralizes with high efficiency both extracellular and intracellular bacterial cell wall-derived toxins, such as LPS and LP. This property is connected with a conformational change of the toxins (aggregate structure, fluidity of their acyl chains, surface charges) converting them into a bioinactive conformation. The peptide also counteracts the pro-inflammatory activity of endotoxin released by antibiotics *in vivo* and cooperates with conventional antimicrobials to reduce inflammation caused by bacterial infections. This is accompanied by a disturbance of the bacterial membranes, which enhances the activity of conventional antibiotics. Thus, this compound in combination with antibiotics could be a life-saving contribution for the treatment of sepsis and other infectious diseases.

## Author Contributions

LH, GW, GM-d-T, and KB conceived and wrote the manuscript. WC, ThG, PG, SS-G, SB-V, and ToG edited the manuscript. All the authors read and approved the final manuscript.

## Conflict of Interest Statement

The authors declare that the research was conducted in the absence of any commercial or financial relationships that could be construed as a potential conflict of interest.

## References

[1] Lepper PM Held TK Schneider EM Bolke E Gerlach H Trautmann M. Clinical implications of antibiotic-induced endotoxin release in septic shock. Intensive Care Med (2002) 28:824–33. 10.1007/s00134-002-1330-6 12122518

[2] Poirel L Jayol A Nordmann P. Polymyxins: antibacterial activity, susceptibility testing, and resistance mechanisms encoded by plasmids or chromosomes. Clin Microbiol Rev (2017) 30:557–96. 10.1128/CMR.00064-16 28275006 PMC5355641

[3] Brandenburg K Heinbockel L Correa W Lohner K. Peptides with dual mode of action: killing bacteria and preventing endotoxin-induced sepsis. Biochim Biophys Acta (2016) 1858:971–9. 10.1016/j.bbamem.2016.01.011 26801369

[4] Heinbockel L Sanchez-Gomez S Martinez de Tejada G Domming S Brandenburg J Kaconis Y Preclinical investigations reveal the broad-spectrum neutralizing activity of peptide Pep19-2.5 on bacterial pathogenicity factors. Antimicrob Agents Chemother (2013) 57:1480–7. 10.1128/AAC.02066-12 23318793 PMC3591871

[5] Schuerholz T Doemming S Hornef M Martin L Simon TP Heinbockel L The anti-inflammatory effect of the synthetic antimicrobial peptide 19-2.5 in a murine sepsis model: a prospective randomized study. Crit Care (2013) 17:R3. 10.1186/cc11920 23302299 PMC4057408

[6] Barcena-Varela S Martinez-de-Tejada G Martin L Schuerholz T Gil-Royo AG Fukuoka S Coupling killing to neutralization: combined therapy with ceftriaxone/Pep19-2.5 counteracts sepsis in rabbits. Exp Mol Med (2017) 49:e345. 10.1038/emm.2017.75 28620220 PMC5519016

[7] Vandamme D Landuyt B Luyten W Schoofs L A comprehensive summary of LL 37, the factotum human cathelicidin peptide. Cell Immunol (2012) 280:22–35. 10.1016/j.cellimm.2012.11.009 23246832

[8] Scott A Weldon S Buchanan PJ Schock B Ernst RK McAuley DF Evaluation of the ability of LL-37 to neutralise LPS in vitro and ex vivo. PLoS One (2011) 6:e26525. 10.1371/journal.pone.0026525 22028895 PMC3196584

[9] Gordon YJ Huang LC Romanowski EG Yates KA Proske RJ McDermott AM. Human cathelicidin (LL-37), a multifunctional peptide, is expressed by ocular surface epithelia and has potent antibacterial and antiviral activity. Curr Eye Res (2005) 30:385–94. 10.1080/02713680590934111 16020269 PMC1497871

[10] Cytrynska M Mak P Zdybicka-Barabas A Suder P Jakubowicz T. Purification and characterization of eight peptides from *Galleria mellonella* immune hemolymph. Peptides (2007) 28:533–46. 10.1016/j.peptides.2006.11.010 17194500

[11] Correa W Manrique-Moreno M Patiño E Peláez-Jaramillo C Kaconis Y Gutsmann T *Galleria mellonella* native and analogue peptides Gm1 and ΔGm1. I) Biophysical characterization of the interaction mechanisms with bacterial model membranes. Biochim Biophys Acta (2014) 1838:2728–38. 10.1016/j.bbamem.2014.07.006 25017800

[12] Correa W Manrique-Moreno M Behrends J Patiño E Marella C Peláez-Jaramillo C *Galleria mellonella* native and analogue peptides Gm1 and ΔGm1. II) Anti-bacterial and anti-endotoxic effects. Biochim Biophys Acta (2014) 1838:2739–44. 10.1016/j.bbamem.2014.07.005 25016054

[13] Lakshmikanth CL Jacob SP Chaithra VH de Castro-Faria-Neto HC Marathe GK. Sepsis: in search of cure. Inflamm Res (2016) 65:587–602. 10.1007/s00011-016-0937-y 26995266

[14] Fink MP Warren HS. Strategies to improve drug development for sepsis. Nat Rev Drug Discov (2014) 13:741–58. 10.1038/nrd4368 25190187

[15] Bone RC Balk RA Fein AM Perl TM Wenzel RP Reines HD A second large controlled clinical study of E5, a monoclonal antibody to endotoxin: results of a prospective, multicenter, randomized, controlled trial. The E5 Sepsis Study Group. Crit Care Med (1995) 23:994–1006. 10.1097/00003246-199506000-00003 7774238

[16] McCloskey RV Straube RC Sanders C Smith SM Smith CR. Treatment of septic shock with human monoclonal antibody HA-1A. A randomized, double-blind, placebo-controlled trial. CHESS Trial Study Group. Ann Inter Med (1994) 121:1–5. 10.7326/0003-4819-121-1-199407010-00001 8198341

[17] Pfalzgraff A Heinbockel L Su Q Gutsmann T Brandenburg K Weindl G. Synthetic antimicrobial and LPS-neutralising peptides suppress inflammatory and immune responses in skin cells and promote keratinocyte migration. Sci Rep (2016) 6:31577. 10.1038/srep31577 27509895 PMC4980674

[18] Yang J Zhao Y Shao F. Non-canonical activation of inflammatory caspases by cytosolic LPS in innate immunity. Curr Opin Immunol (2015) 32:78–83. 10.1016/j.coi.2015.01.007 25621708

[19] Kayagaki N Warming S Lamkanfi M Vande Walle L Louie S Dong J Non-canonical inflammasome activation targets caspase-11. Nature (2011) 479:117–21. 10.1038/nature10558 22002608

[20] Pfalzgraff A Heinbockel L Su Q Brandenburg K Weindl G. Synthetic anti-endotoxin peptides inhibit cytoplasmic LPS-mediated responses. Biochem Pharmacol (2017) 140:64–72. 10.1016/j.bcp.2017.05.015 28539262

[21] Khare S Dorfleutner A Bryan NB Yun C Radian AD de Almeida L An NLRP7-containing inflammasome mediates recognition of microbial lipopeptides in human macrophages. Immunity (2012) 36:464–76. 10.1016/j.immuni.2012.02.001 22361007 PMC3315380

[22] Vanaja SK Russo AJ Behl B Banerjee I Yankova M Deshmukh SD Bacterial outer membrane vesicles mediate cytosolic localization of LPS and caspase-11 activation. Cell (2016) 165:1106–19. 10.1016/j.cell.2016.04.015 27156449 PMC4874922

[23] Gutsmann T Razquin-Olazaran I Kowalski I Kaconis Y Howe J Bartels R New antiseptic peptides to protect against endotoxin-mediated shock. Antimicrob Agents Chemother (2010) 54:3817–24. 10.1128/AAC.00534-10 20606063 PMC2934961

[24] Kaconis Y Kowalski I Howe J Brauser A Richter W Razquin-Olazaran I Biophysical mechanisms of endotoxin neutralization by cationic amphiphilic peptides. Biophys J (2011) 100:2652–61. 10.1016/j.bpj.2011.04.041 21641310 PMC3117184

[25] Garidel P Brandenburg K Current understanding of polymyxin B applications in bacteraemia/sepsis therapy prevention: clinical, pharmaceutical, structural and mechanistic aspects. Anti-Inf Agents Med Chem (2009) 8:367–85. 10.2174/187152109789760171

[26] Heinbockel L Palacios-Chaves L Alexander C Rietschel E Behrends J Correa W Mechanism of Hbgamma-35-induced an increase in the activation of the human immune system by endotoxins. Innate Immun (2015) 21:305–13. 10.1177/1753425914535957 25034969

[27] Seydel U Koch MHJ Brandenburg K. Structural polymorphisms of rough mutant lipopolysaccharides Rd to Ra from *Salmonella minnesota*. J Struct Biol (1993) 110:232–43. 10.1006/jsbi.1993.1026 8373704

[28] Brandenburg K Heinbockel L Correa W Fukuoka S Gutsmann T Zähringer U Supramolecular structure of enterobacterial wild-type lipopolysaccharides (LPS), fractions thereof, and their neutralization by Pep19-2.5. J Struct Biol (2016) 194:68–77. 10.1016/j.jsb.2016.01.014 26828112

[29] Jiao BH Freudenberg M Galanos C. Characterization of the lipid A component of genuine smooth-form lipopolysaccharide. Eur J Biochem (1989) 180:515–8. 10.1111/j.1432-1033.1989.tb14676.x 2714268

[30] Howe J Andra J Conde R Iriarte M Garidel P Koch MH Thermodynamic analysis of the lipopolysaccharide-dependent resistance of Gram-negative bacteria against polymyxin B. Biophys J (2007) 92:2796–805. 10.1529/biophysj.106.095711 17237210 PMC1831710

[31] Martinez de Tejada G Heinbockel L Ferrer-Espada R Heine H Alexander C Barcena-Varela S Lipoproteins/peptides are sepsis-inducing toxins from bacteria that can be neutralized by synthetic anti-endotoxin peptides. Sci Rep (2015) 5:14292. 10.1038/srep14292 26390973 PMC4585737

